# Comparing the Clinical Viability of Automated Fundus Image Segmentation Methods

**DOI:** 10.3390/s22239101

**Published:** 2022-11-23

**Authors:** Gorana Gojić, Veljko B. Petrović, Dinu Dragan, Dušan B. Gajić, Dragiša Mišković, Vladislav Džinić, Zorka Grgić, Jelica Pantelić, Ana Oros

**Affiliations:** 1 The Institute for Artificial Intelligence Research and Development of Serbia, 21102 Novi Sad, Serbia; 2 Faculty of Technical Sciences, University of Novi Sad, 21102 Novi Sad, Serbia; 3 Eye Clinic Džinić, 21107 Novi Sad, Serbia; 4 Institute of Eye Diseases, University Clinical Center of Serbia, 11000 Belgrade, Serbia; 5 Institute of Neonatology, 11000 Belgrade, Serbia

**Keywords:** fundus image, segmentation, segmentation mask, subjective assessment, convolutional neural networks, clinical viability, objective metrics, subjective metrics

## Abstract

Recent methods for automatic blood vessel segmentation from fundus images have been commonly implemented as convolutional neural networks. While these networks report high values for objective metrics, the clinical viability of recovered segmentation masks remains unexplored. In this paper, we perform a pilot study to assess the clinical viability of automatically generated segmentation masks in the diagnosis of diseases affecting retinal vascularization. Five ophthalmologists with clinical experience were asked to participate in the study. The results demonstrate low classification accuracy, inferring that generated segmentation masks cannot be used as a standalone resource in general clinical practice. The results also hint at possible clinical infeasibility in experimental design. In the follow-up experiment, we evaluate the clinical quality of masks by having ophthalmologists rank generation methods. The ranking is established with high intra-observer consistency, indicating better subjective performance for a subset of tested networks. The study also demonstrates that objective metrics are not correlated with subjective metrics in retinal segmentation tasks for the methods involved, suggesting that objective metrics commonly used in scientific papers to measure the method’s performance are not plausible criteria for choosing clinically robust solutions.

## 1. Introduction

As the world population ages, the number of people suffering from impaired vision increases [1]. This trend is poised to continue since healthcare development prolongs life expectancy, and this, in turn, increases the prevalence of age-related eye diseases, such as glaucoma, and age-related macular degeneration. Added to other eye diseases that may occur in adulthood or childhood, such as diabetic retinopathy, hypertensive retinopathy, and retinopathy of prematurity, it is evident that early diagnosis and treatment of this degenerative class of eye diseases is essential in improving the overall quality of life.

**Figure 1:**
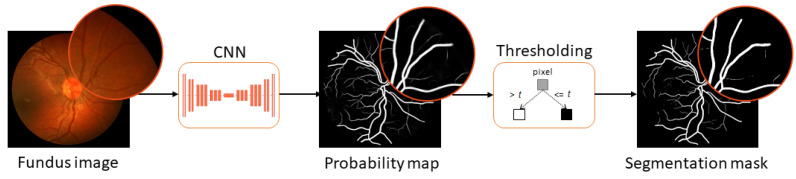
A typical retinal vessel segmentation workflow consists of probability map inference from an input fundus image followed by thresholding *t* to create a binary segmentation mask from the probability map.

An important diagnostic resource in eye disease treatment and screening is the fundus image, such as the one shown in Figure 1 (the image on the left). During the screening, a trained expert observes the fundus image looking for early signs of disease. This process is often hampered by the low-contrast nature of fundus images, as well as image artifacts, such as blur and focus issues, that can appear in clinical practice, inevitably leading to mistakes in diagnosis. With a rapidly aging population and a proportional deficit in the number of experts, the workload per expert is increasing, increasing the chance of a misdiagnosis due to human error. This motivated the development of automated methods for fundus image vessel segmentation which would aid experts by automating image analysis and then eventually, replacing expert involvement, especially in early screening [2].

Most recent research on automatic vessel segmentation in fundus images uses convolutional neural networks (CNNs). This is a class of deep learning methods most commonly applied to analyze images. Convolutional neural networks have already demonstrated their superiority over traditional methods in a variety of medical image segmentation applications, such as lesion [3,4,5], organ [6,7,8,9], cell [10,11], and tumor [12,13] segmentation from different image modalities, such as computed tomography (CT) or magnetic resonance imaging (MRI). Figure 1 shows how CNN is used in fundus image segmentation. As an input, CNN accepts a color fundus image and outputs a probability map, typically represented as a grayscale image where the color intensity of each pixel in the map reflects the probability that the corresponding pixel in the input image represents a blood vessel. This means that black pixels in the map indicate 0% probability that the corresponding pixel in the input fundus image represents a blood vessel, while white pixels are considered to represent a blood vessel with 100% probability. Pixels in shades of gray represent varying probabilities between 0% and 100%. Probability maps are usually post-processing to produce segmentation masks, which are black-and-white images where each pixel can be either black to indicate the background or white to indicate the blood vessel pixel in the input fundus image. To produce a segmentation mask, the probability map is processed so that all pixels with a value beneath a certain threshold are set to black and all other pixels are set to white. This process is referred to as ’thresholding’ the image.

In the literature, segmentation masks are the output used to evaluate network accuracy [14]. Here it is common to report objective metrics, such as segmentation accuracy, sensitivity, specificity, F1 score, and Matthews Correlation Coefficient (MCC). All of these are full-reference, pixel-wise metrics, meaning that they are calculated based on pixels of the produced segmentation mask and a ground truth image for a given input fundus image. While it is a good choice to use these metrics for inter-method comparison since they produce deterministic values for constant input, it has been demonstrated in other medical applications that they are not necessarily good approximations of the method’s clinical viability [15]. For example, a CNN can obtain high classification accuracy while still being useless in diagnostics. For instance, it could detect most of the mask correctly and only lose a small proportion of total pixels, while those pixels are the ones most important for a clinician. This problem is particularly noticeable in retinal vessel segmentation since vessel pixels often occupy under 10% of an image while the rest are background pixels. In this case, a naive algorithm that classifies all pixels as background achieves the high accuracy measurement of approximately 90% while simultaneously not producing any clinically meaningful results. This problem is well known in the machine learning literature as a class imbalance problem, where one of the classes in a dataset is underrepresented compared to other classes [16].

The shortcomings of objective evaluation metrics are alleviated through subjective quality assessment studies, where human participants are asked to perform the evaluation. This form of evaluation is suitable for clinical viability studies since errors that have a low impact pixel-wise while still obscuring vital diagnostic details would be poorly rated by human evaluators. Since subjective studies are time-consuming and require human involvement, they are generally harder to perform. Thus they are not as widespread in the literature as objective evaluation studies. In related work, subjective assessment methods in ophthalmology are commonly implemented as a part of a broader evaluation of newly developed automated diagnostic methods. Applications include, but are not limited to, glaucoma detection from stereoscopic [17,18,19] and monoscopic [20] optical disk images, diabetic retinopathy [21,22], diabetic macular edema classification [23], age-related macular degradation [19,22,24,25,26], central/branch retinal vein occlusion [22], detachment of the retina [22], as well as cataract [21,27], retinopathy of prematurity classification [28], and cardiovascular disease detection [29]. Related studies address disease classification tasks, where both human observers and proposed artificial intelligence (AI) methods take the same piece of data as an input and output the same kind of information: a textual diagnosis, a bounding box marking a region of interest where a certain pattern is observed in an image, etc. Having the same output type in these studies makes comparison between proposed automatic methods and human observers straightforward to implement. For the vessel segmentation task, it is not feasible to use this direct comparison protocol due to the nature of segmentation masks that are quickly produced by an algorithm but are hard to be manually annotated by a human since annotation is a time-consuming process requiring per-pixel precision. This is why we deviate from the comparison frameworks present in the literature and propose that the observers evaluate the outputs of the selected CNNs instead of creating outputs that would be directly compared to CNN outputs. In this approach, the observers are shown CNN-generated segmentation masks, asked to choose a diagnosis, and provide quantitative information on the diagnostic quality of the network’s output that is later used to compute an overall ranking. To the best of the authors’ knowledge, this is the first study that addresses the clinical viability of automatically produced retinal blood vessel segmentation masks.

This research is motivated by the inadequacy of objective metrics that represent the diagnostic significance of segmentation masks produced by methods for automatic vessel segmentation of fundus images. We conduct a pilot study to test two hypotheses: (1) automatically generated segmentation masks contain enough clinically valuable information for the diagnosis of diseases affecting the retinal vascularization network, and (2) a ranking of clinical applicability between the tested methods can be established. Both hypotheses are tested through a subjective assessment study implemented as two surveys. The experimental results show that, at present, the quality of automatically produced segmentation masks is insufficient for standalone clinical diagnosis. Although the first hypothesis was rejected, partial confirmation of the second hypothesis indicates that segmentation masks might gain more clinical significance in the future with further improvement of vascularization segmentation methods.

The rest of the paper is structured as follows. In Section 2, we explain how the data used in the subjective assessment is generated, the survey design choices made, as well as the subjective assessment protocol and methods used to analyze and discuss the collected raw results. Section 3 presents the analysis and discussion of the obtained results. The final section presents the main conclusions and suggests further research directions.

## 2. Materials and Methods

This section covers the experimental setup in detail. It explains the process of generating an evaluation dataset and the surveys that are presented to observers through a subjective assessment study. Five experienced ophthalmologists trained in the screening and treatment of ocular diseases were asked to participate in the subjective assessment. Two of the five are specialized in the diagnosis of eye vascular diseases in adults, such as diabetic retinopathy, another two in retinopathy of prematurity in children ophthalmology, and one in all of the above. Four observers have between 10 and 20 years of clinical experience, while the remaining one has more than 30. Results collected in the subjective assessment are then analyzed in terms of descriptive statistics and robust statistical analysis.

### 2.1. Convolutional Neural Networks

**Figure 2:**
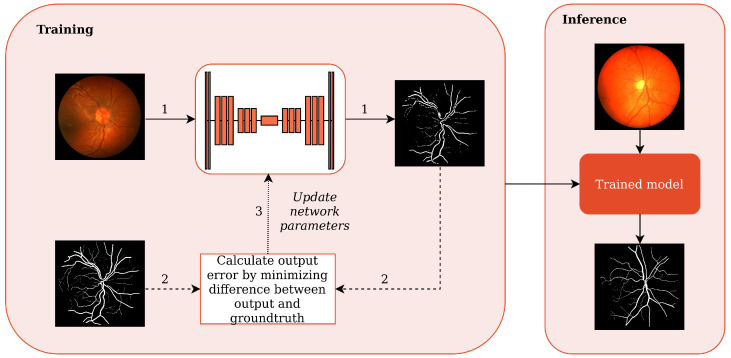
The CNN lifecycle. The training schema shows (1) feeding CNN with training images to obtain predicted outputs, (2) using predicted outputs and ground truth images to update CNN parameters, and (3) updating CNN parameters. In the inference schema, a trained model is fed with unseen images to obtain predictions without the ground truth being known.

Convolutional neural networks are a class of deep learning methods designed to learn the spatial dependencies of grid-like data, such as images. A typical CNN consists of multiple stacked layers that learn to extract spatially significant features from input data. The choice and number of layers in the network define the architecture of the CNN and its capability to learn. Learning is performed through a training process during which the CNN is provided with training data consisting of inputs and their corresponding solutions (the ’ground truth’). Using some form of gradient descent, CNNs are adjusted to match the ground truth output for the corresponding input using an iterative process of minimizing the ’error,’ which is the difference between the network output and the corresponding ground truth. A diverse and deep training dataset permits the network to ’learn’ patterns in the data which it will then generalize. This generalization is verified using cross-validation, i.e., measuring network performance on data it has never ’seen.’ The output of the training process is a trained model with its structure and weights fixed and able to perform inference over arbitrary input data. The whole pipeline is shown in Figure 2. For a comprehensive overview of CNN foundations, the reader is referred to [30].

In this study, we have selected seven convolutional neural networks for blood vessel segmentation that reports high segmentation performance in terms of objective metrics and have publicly available source code for network training and inference, making the experiment reproducible. The selected networks are SA-UNet [31], IterNet [32], LadderNet [33], UNet [34], ESWANet [35], and two networks for which we have coined the names RVSGAN [36] and DBUNet [37]. Architecturally, these networks are variations of the UNet architecture that is commonly used in medical image segmentation since it can be trained with moderately small datasets and still produce satisfactory results. All networks except ESWANet build on the UNet architecture either by chaining multiple networks [32,33], substituting some of the default UNet building blocks with more efficient mechanisms [17,31,34,35] or using UNet as a part of a more complex network, such is the case in Generative Adversarial Networks (GANs) [36].

UNet is a basic architecture for medical image segmentation designed to learn from small datasets and utilize data augmentation. The architecture consists of an encoder for cascaded image feature extraction and dimensionality reduction and a decoder that uses features learned by the encoder to gradually reconstruct the segmentation probability map. Repeated use of convolution and max pooling layers in the encoder enables the network to extract local and global features from input data. In the decoder branch, upsampling and convolution are used repetitively to reconstruct the segmentation mask from learned features. Learning is facilitated by skip connections directly providing learned features from the encoder layer to the corresponding decoder layer. DBUNet replaces the standard UNet blocks with residual ones to alleviate vanishing and exploding gradient problems and improve simple pattern learning. SA-UNet introduces a spatial attention module into the UNet architecture to learn spatial maps used to refine features adaptively. The architecture replaces the standard UNet convolutional block with a structured dropout convolutional block to speed up training convergence and reduce the number of training parameters. Inspired by the idea that humans annotate fundus images in multiple passes to obtain detailed segmentation masks and correct errors, the IterNet network employs multiple chained UNet networks to predict a coarse segmentation mask and then refine it. LadderNet chains multiple UNet architectures by connecting corresponding layers of neighboring decoders and encoders, introducing additional information flow paths leveraging learning. To reduce the number of parameters introduced by new connections, the author uses residual blocks consisting of two convolutional layers with weight-sharing, batch normalization, and dropout. ESWANet is similar to UNet since it consists of encoder and decoder branches introducing skip connections between layers and featuring concatenation. It uses max pooling and cropping to reduce feature map size and upsampling to increase the dimensionality of feature maps. Unlike UNet, it does not have a symmetrical, "U" shape structure. RVSGAN is a generative adversarial network consisting of a generator and discriminator jointly trained to generate a segmentation mask. A generator is trained to produce a segmentation mask for an input image, while the discriminator is trained to distinguish between the generator and ground truth segmentation masks. As a generator, RVSGAN uses the UNet architecture, while the discriminator is a CNN encoder with a fully connected layer and sigmoid activation. The training hyperparameters of the methods used in the experiment are displayed in Table 1.

To generate the segmentation masks required for the observers to make the assessment, trained models for each of the networks of the DRIVE, STARE, and CHASE datasets are necessary. In most cases, we have used publicly available pre-trained models provided by the authors, with the exception of the RVSGAN network and the CHASE dataset. For this particular case, we have trained the model on the CHASE dataset using a modified training script compared to the one provided by the authors. The reason for the use of the modified script is the performance impact of using the CHASE dataset with the original script. We believe this performance issue was the reason that the authors have provided models just for the DRIVE and STARE datasets since they also explicitly warn of the high computational demands of the training script. The modified script uses randomly selected image patches 128×128 pixels in size, with 64 patches per batch, instead of the whole image as in the original script to train the network. The training is performed for 10 epochs, 4800 batches per epoch, with hyperparameters set to values reported in the original paper for both the discriminator and generator.

This all resulted in 21 trained models produced by training each of the 7 CNN architectures for each of the 3 datasets. We added one more additional model provided by the authors for the IterNet network pretrained on all images in the DRIVE, STARE, and CHASE datasets. Later in the paper, we refer to this model as IterNet-UNI, while other models are named following the pattern network_name-dataset_name.

### 2.2. Evaluation Dataset

The evaluation dataset used in the subjective assessment consists of color retinal images from DRIVE, STARE, and CHASE datasets, as well as corresponding segmentation masks generated by all eight selected CNN models for each color fundus image.

#### 2.2.1. Base Dataset

**Figure 3:**
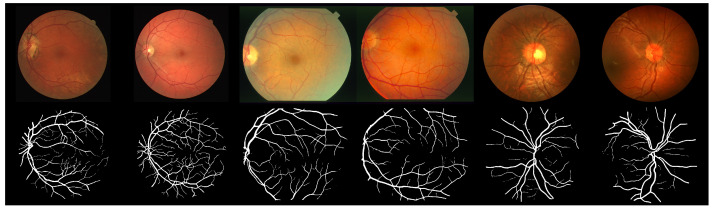
Examples of images used in the study. In the first row are fundus images from DRIVE, STARE, and CHASE datasets. In the second row are the corresponding segmentation masks.

Fundus images from DRIVE, STARE, and CHASE public databases are used as a basis for creating evaluation datasets. When combined, these datasets provide a rich fundus image database in terms of disease diagnosis, with images showing signs of diabetic retinopathy, arteriosclerotic retinopathy, etc. Examples of the images are shown in Figure 3 in the first row.

The Digital Retinal Images for Vessel Extraction (DRIVE for short) dataset [38] contains 40,768 × 584 px color fundus images collected as a part of a Netherlands-based screening trial for diabetic retinopathy. The images are captured using a 3CCD camera with a 45${}^{\circ }$ field of view (FoV). Seven images demonstrate mild signs of diabetic retinopathy, while the others have no pathology signs.

The Structured Analysis of Retina (STARE for short) dataset [39] consists of 20,700 × 605 px color fundus images captured with a Topcon TRV-50 fundus camera with a 35${}^{\circ }$ FoV. Among those, 10 images show signs of diabetic retinopathy, choroidal neovascularization, arteriosclerotic retinopathy, and different forms of artery and vein occlusions, while the remaining 10 are shots of healthy adult retinas.

A subset of 28 retinal images collected in a Child Heart and Health Study in England (abbreviated to CHASE) [40] make up the CHASE dataset. The survey was performed to investigate possible associations between blood vessel tortuosity and risk factors for cardiovascular diseases in children. The images are captured with a 30${}^{\circ }$ FoV Nidek NM-200-D fundus camera with a resolution of 1280×960 px. There is no pathology information associated with the images provided in this dataset.

#### 2.2.2. Generating Segmentation Masks

**Figure 4:**
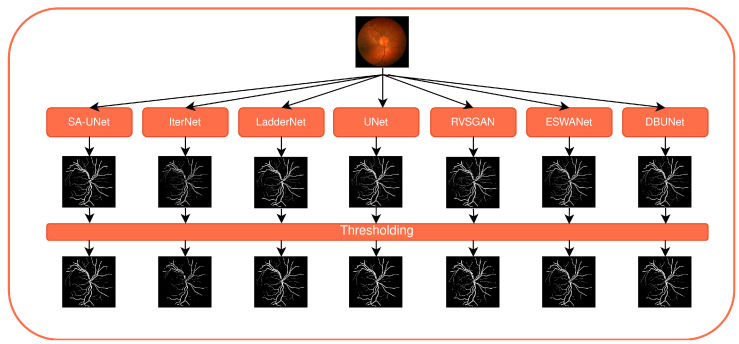
Experimental pipeline illustrating a pipeline to create segmentation masks used in the experiment from input fundus images.

A pipeline to predict segmentation masks for an input image database is presented in Figure 4. Each fundus image from the base dataset is fed to each of the trained models to produce a corresponding probability map. Each probability map is then thresholded with a value of 0.5, meaning that each pixel of a probability map with an intensity lower than 0.5 is treated as a background pixel, while each pixel of intensity equal to or above 0.5 is treated as a vessel pixel. The final dataset used in subjective assessments consists of 704 segmentation masks, equaling 88 masks per model or 8 different segmentation masks per each fundus image produced by 8 different models. Examples of segmentation masks are shown in the second row of Figure 3.

### 2.3. Study Design

Subjective assessment is carried through in two stages. The first stage aimed to gain insight into the clinical viability of segmentation masks produced by AI methods. Specifically, it aimed to deduce how much diagnostic information can be extracted solely from segmentation masks. Stage two attempts to differentiate between various networks in their clinical applicability and aims to produce a ranking and also some insight into the interrelation of architectural features of networks and their clinical applicability. Both assessments are implemented as online surveys using the SurveyJS framework [41]. All observers have completed the first survey, while four of them, including the most experienced one, have completed the second survey.

#### 2.3.1. Stage 1: Assessing Diagnostic Significance of Segmentation Masks

The purpose of this stage is to assess if segmentation masks produced by automated fundus segmentation techniques can be used as a standalone resource for diagnostic purposes and, if so, how successful the observers are in diagnostics from this imaging modality. To determine this, each of the observers has received a unique link to the series of eight online surveys. The same surveys are shown to all the observers in the same order. The observers are asked to fill out the surveys independently, but no other restriction is placed on the circumstances or environment of their work. This choice was made to mimic real-world working conditions in which results produced by the automated AI method would be interpreted.

In each survey, an observer is presented with a sequence of 15 questions, each displaying a test image and asking the observer to provide two answers related to that test image. The test image is a segmentation map the observer is meant to use as the basis for diagnosis. The two ratings are a diagnosis (selected from a list) and confidence in that diagnosis. The observers can also mark the image as completely unsuitable for diagnosis. Details on the survey design are described in Appendix A.

The last two surveys from the series are composed solely of randomly selected questions from the first six surveys. This is performed to check intra-observer consistency.

Table 2 shows the list of fundus images whose segmentation masks are presented to the observers through survey questions. As can be seen in the table, we have included a subset of images from the image database originating solely from DRIVE and STARE datasets. These images are selected since, according to diagnostic data provided by the dataset authors, they demonstrate signs of pathology. While it is known that the CHASE dataset was collected in retinopathy of prematurity screening trial, an exact list associating particular images from the dataset with diagnostic labels was not provided by the authors, making the images from this dataset unsuitable for this experiment due to a lack of exact per-image diagnoses. Images presented to the observers show signs of background diabetic retinopathy, arteriosclerotic retinopathy, cilio-retinal artery occlusion, and central retinal artery and vein occlusion. Some of the images are associated with multiple diagnoses. In that case, should the observer select any of the listed diagnoses, the answer will be marked as correct. For purposes of the experiment, central retinal artery occlusion and central retinal vein occlusion are aggregated into a single central retinal artery/vein occlusion diagnosis. Aggregation is performed due to the nature of segmentation masks: at this stage of development, they do not differentiate between arteries and veins, identifying only blood vessels of any type from the background. Further, background diabetic retinopathy, a subclass of diabetic retinopathy, is treated as diabetic retinopathy to have uniform nomenclature over DRIVE and STARE datasets.

Among segmentation masks shown to the experts in the first phase, there were no segmentation masks produced by the UNet model since these masks were accidentally omitted in the survey generation process. We were unable to correct this issue since the observers involved in the study were not available to fill out additional surveys when the omission was spotted. However, since the other CNNs involved are UNet variations demonstrating consistent trends, as discussed in Section 3, the pure UNet architecture would likely perform similarly to its advanced successors, so we do not consider this a major issue for the overall research.

#### 2.3.2. Stage 2: Ranking CNNs According to Clinical Viability

Although CNNs used in medical image diagnosis are usually based on UNet architectures, some architectural variations might perform better than others for the particular task of retinal vessel segmentation. The purpose of the second experiment is to test if the ranking between selected networks can be established. This knowledge could be useful in directing the design of future CNN architectures for blood vessel segmentation toward those that excel and away from underperforming ones.

**Figure 5:**
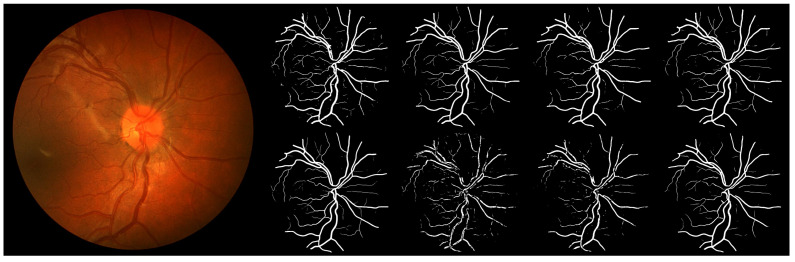
An example of a color fundus image and corresponding eight segmentation masks produced by eight CNN models. From left to right, the masks are generated by DBUNet, RVSGAN, UNet, SA-UNet (top row), LadderNet, IterNet-UNI, IterNet, and ESWANet (bottom row).

Each of the observers has been granted access to 75 surveys. The observers are asked to fill out the surveys independently in the environment they work in. In each survey, the observers rank eight segmentation masks for the same fundus image, each produced by a different CNN model. As can be seen in Figure 5, the segmentation masks produced by different models are similar in appearance, which makes it tedious to rank all eight in a single pass. To simplify the process, the comparison is implemented as a series of pairwise comparisons, where the observer is presented with a fundus image for reference and two segmentation masks that are being compared. Each pairwise comparison is a survey question where the observer chooses one of the two segmentation masks they find to be diagnostically more valuable. The survey contains 42 questions, where 28 show image pairs produced as combinations without repetition for the starting 8 segmentation masks with a sampling rate of 2, and the rest are randomly sampled questions from the first 28 in order to provide data for intra-observer consistency calculations. Details on the survey design are shown in Appendix A.

### 2.4. Statistical Analysis

To analyze the results collected in the first and second experiments, we first calculate scores for each of the answers from both surveys. In the first experiment, the score or, henceforth, *grade* is calculated as a function of answer correctness and observer certainty in a given answer. To calculate the grade we use the expression is_correct·certainty where is_correct∈{1, −1, 0} for correct diagnosis, incorrect diagnosis, and not applicable for diagnosis, respectively, and certainty∈[1, 5] forming a Likert scale from uncertain to completely certain. This means that the *grade* will be positive for a correct diagnosis with a maximum value of 5 when the observer is completely certain of a given response or negative for an incorrect answer with a minimal value of −5 when the observer is completely certain of a decision.

Copeland’s voting algorithm [42] is used to rank networks in the second experiment. This method was selected due to the design of the second survey, where a group of segmentation masks is compared using multiple pairwise comparisons. A pseudo-code to calculate the Copeland score is shown in Appendix B.

Both scores were subject to reliability analysis using Cronbach’s α and Guttman’s $\lambda_{6}$ using the methods of Feldt, Dunhachek, and iterative bootstrapping with n=2000. For the first experiment, the reliability measure is computed on original and repeated measurements. For the second experiment, the reliability measure is computed on the original ranking of better/worse, and the same question is repeated at a later date. In both cases, the observers were not informed of the presence of control questions. These analyses are used to determine if the results are reliable, i.e., they estimate the likelihood that, given the same questions again, the observers will give the same responses. A high value indicates a stable opinion, and a low value indicates that it was difficult for observers to reproduce the answer to the same question.

For further analysis of the grade from the first experiment, we use a subvariant of Yuen’s test with 20% trimmed means that take into account the dependent nature of the samples, which is a robust analog to a pooled variance-dependent sample *t*-test. To measure the effect size, we used a robust measure of effect size for dependent samples called the Algina–Keselman–Penfield effect size [43], which is a robust analog to Cohen’s *d*. In both cases, we want relatively simple analyses but are forced to resort to robust statistical methods, which produce the same results as the simple methods but are resilient to false readings due to small sample sizes or violations of the conditions of normality or independence of residuals.

## 3. Results and Discussion

The statistical tests were analyzed using the R statistical analysis environment [44], version 4.1.3, 64-bit, running on Fedora Workstation 36 Linux. Analysis was conducted with the aid of the Psych [45], IRR [46], and WRS2 [47] packages.

### 3.1. Stage 1: Assessing Diagnostic Significance of Segmentation Masks

**Figure 6:**
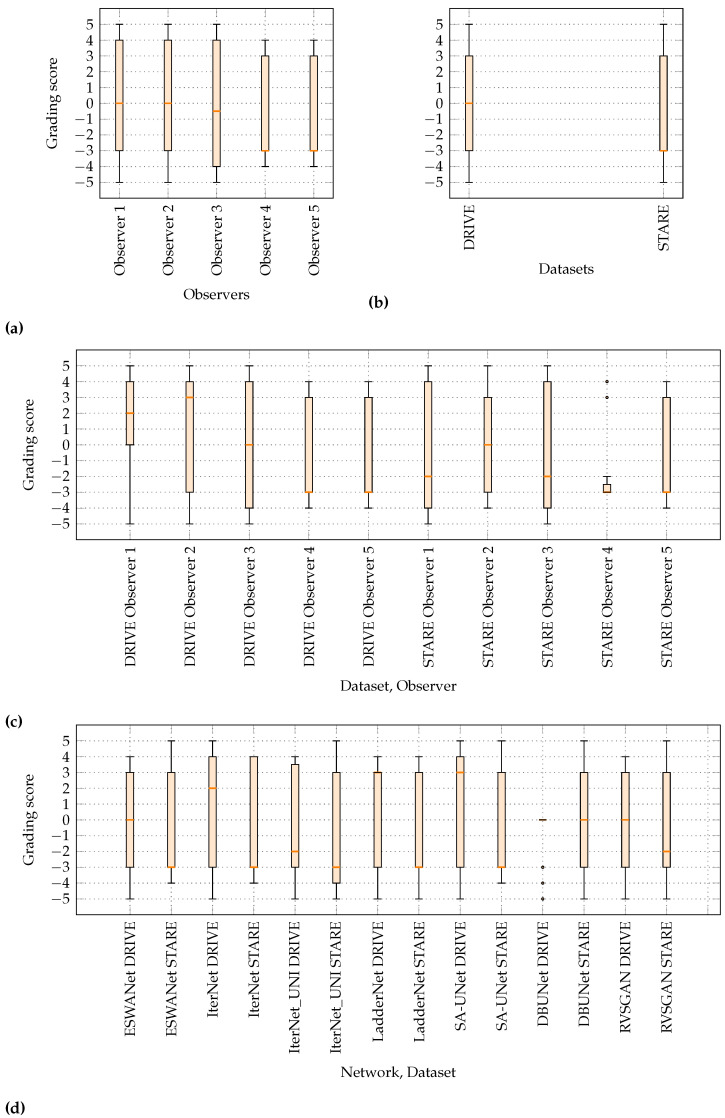
Grading score distributions grouped by (**a**) observers, (**b**) datasets, (**c**) datasets and observers, and (**d**) networks and observers. The elementary results for UNet-generated segmentation masks were not included for the reasons discussed in Section 2.3.1.

Figure 6a compares the grading score distributions for all observers involved in the study obtained from regular surveys. The distributions are nearly uniform (Observers 1, 2, and 3), meaning that the observers have assigned a segmentation mask with a correct diagnosis in approximately half of the cases when presented with seven different choices or skewed (Observers 4 and 5) with an overall accuracy below 50%. The observers had more success in inferring correct diagnoses from DRIVE than STARE originating segmentation masks, as is shown in Figure 6b. For the DRIVE dataset, the grading scores are distributed uniformly over the whole grading score range, with an equal number of scores for correct and incorrect diagnoses. For the STARE dataset, more than half of the score samples are negative, meaning that observers were more likely to make a mistake given a segmentation mask originating from STARE in general. A pattern of DRIVE scoring higher than STARE is consistent when comparing both observer and network distributions. For observer comparison, this is visible in Figure 6c where all distributions related to STARE are positively skewed, and most of the DRIVE-related distributions are negatively skewed. The same is true for network comparison, as shown in Figure 6d. Further analysis of the classification accuracy for each of the disease classes (Table 3) demonstrates that not all diseases are equally successfully diagnosed from segmentation masks. Images showing signs of cilio-retinal artery occlusion and arteriosclerotic retinopathy present in the STARE dataset lead to overall lower classification accuracy on the dataset with an educated guess that segmentation masks do not express enough vascularization structural deformations to diagnose these diseases solely from segmentation masks. The observers were more successful in diagnosing diabetic retinopathy and central retinal/vein occlusion from segmentation masks. However, absolute classification accuracy for these two classes indicates the study is on the edge of being clinically possible since the observers are trained to assess diagnosis from color fundus images or that selected CNNs are unable to produce segmentation masks of sufficient quality for diagnosis. This observation was a starting point for the follow-up study to test if the selected methods were capable of producing segmentation masks of consistent quality.

A total of n=570 tests were conducted, and of these, a proportion of 26.32% was repeated as controls to test the intra-observer consistency of the measurements. Values for $\lambda_{6}$ and α coefficients are provided in the first row in Table 4. The first form of analysis was to compute reliability scores for all observers in aggregate. The values obtained indicate that the grading score is an unreliable measure, revealing that observers were not able to reproduce their results a control group of questions. If the reliability analysis is limited to only the most experienced observer who had demonstrated the best accuracy, especially for the DRIVE dataset (see the first box plot in Figure 6c), the resultant measures are still 0.6 and 0.4 for α and $\lambda_{6}$ with a 95% CI for the α of 0.16 to 0.83. This means that not even the best observer can produce reliable results. The results are the same no matter whether we take observer confidence into consideration or not (see the second row of Table 4). The end result is always the same; intra-observer consistency is low.

Based on the reliability measurement, and on the results of robust statistical analysis, the hypothesis that segmentation masks produced by a selected group of CNN models can be used as a standalone resource for clinical diagnosis is rejected. The failure of this hypothesis is supported by both exploratory analysis and robust statistics, confirming that grading scores obtained on regular and control group questions are not repeatable and, in fact, deviate a lot not just in terms of confidence but also in terms of correctness.

### 3.2. Stage 2: Ranking CNNs According to Clinical Viability

A total of n=2856 survey rankings were conducted, and of these, a proportion of 33.33% was repeated as a control to establish the reliability of the measurement. In summary, we have measured a total value of Cronbach’s α of 0.7 and a total value of $\lambda_{6}$ of 0.5 with 95% confidence intervals for the α of 0.66 to 0.75. This indicates a reliable measure, meaning that the doctors had a stable preference for one image over another, even when asked again after considerable time. This means that these results are worth further analysis since they represent a measurable, repeatable opinion of domain experts.

We also conducted a test of inter-rater reliability. Intra-rater reliability shows that, over time, test participants retain their opinions and produce the same results to the same questions. Inter-rater reliability, however, measures how similar the opinions of various observers are, i.e., if there is agreement that everyone produces the same responses to the same questions. We used the Fleiss variation for m raters of Cohen’s κ measure. In this approach, we treated the expert observers as raters, in the Cohen–Fleiss sense, and each answer of a head-to-head comparison of two segmentation masks as them rating one image over another. This rating of one observer over another was treated as a ’subject,’ in the Cohen–Fleiss sense. Redundant tests were omitted. The results are κ=0.130719 with a test statistic of 6.9858244, leading to a *p*-value of $2.8319569\;\cdot \;10^{-12}$. The interpretation here is that there is not a lot of agreement between the experts on ranking: the observers are very certain of their opinion but they do not all share it, possibly due to variations in experience.

Table 5 displays the CNN ranking obtained on all pairwise comparisons for segmentation masks of all three datasets and all observers that took the second experiment. One observer did not finish the experiment in a timely fashion, so we derived results from the data collected from the remaining four observers. When calculating the Copeland score for each network, we employ the +1/0/−1 strategy. This means that networks with a positive Copeland score have won more pairwise comparisons than they have lost. A higher value of the Copeland score means that it is recognized by observers that the network generates segmentation masks of greater clinical quality. According to Table 5, the best results are achieved by RVSGAN, UNet, and LadderNet networks, followed by ESWANet and SA-UNet, with the lowest results for IterNet and DBUNet. Since Table 6 shows a bias toward positive scores for ESWANet, UNet, and RVSGAN consistent on the observer level, it is possible that simple UNet-like architectures and generative adversarial networks containing UNet might be the right architectural choice for the development of retinal vascularization solutions appropriate for clinical usage. We establish this as an assumption that should be tested in detail in further studies that would standardize the network comparison protocol.

In the second experiment, DBUNet ranks the lowest, which matches the patterns noted in the first survey data; see Figure 6d. Table 7 confirms that observers considered the DBUNet segmentation masks of lower clinical quality compared to segmentation masks produced by other networks.

The Copeland method can also be executed on a per-dataset level, producing a factored analysis. In Table 6, it can be observed that DBUNet has the most negative score for the DRIVE dataset, while the overall performance on the STARE and CHASE datasets is positive with moderately high score values. A high negative bias toward the DRIVE dataset can explain the overall low score of the network. Looking at Table 6 and Table 7, it can be seen that there is no strict winner in the Condorcet sense. This means that there is no universally effective network that produces the consistently best result for all observers and all datasets. Given this finding, the best approach toward the clinical utility of the present models would be a combination of multiple CNNs operating in an ensemble fashion. If each CNN provides masks, and if each excels at its specific case, the result should exceed the reliability of any one network. Combining the results in such a way that they are of use to both human diagnostic mechanisms and artificially intelligent ones, however, remains to be researched.

**Figure 7:**
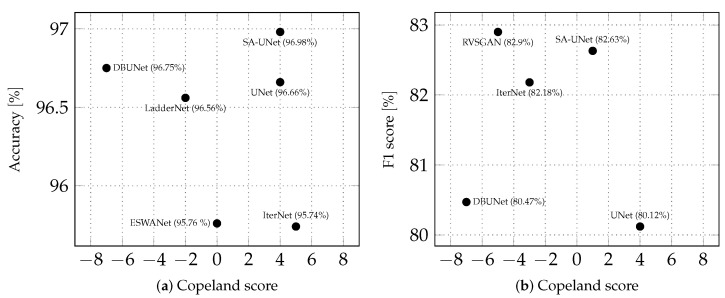
Objective vs. subjective metrics on the DRIVE dataset. The Copeland score is on the *x*-axes, while the *y*-axes is (**a**) accuracy (RVSGAN and IterNet_UNI are ommitted since the original paper does not report corresponding accuracy values) and (**b**) F1 score, respectively (LadderNet and IterNet_UNI are omitted since the original paper does not report corresponding accuracy values). Each dot on a diagram corresponds to one of the CNNs with an associated Copeland score and objective metric value. The accuracy and F1 score values for RSVGAN, ESWANet, SA-UNet, and IterNet are from the original papers. Values for DBUNet are from supplementary materials of the project used in [37]. The results for UNet are from [31] since UNet was not evaluated on the DRIVE dataset in the original paper.

Figure 7 shows how the Copeland score, as a subjective metric, corresponds to the accuracy and F1 score, which are two commonly used objective metrics when evaluating blood vessel segmentation methods. The purpose of the plot is to visually inspect if there is a positive or negative correlation between network ranking and objective metrics for the network. Typically, correlation is also expressed numerically through Pearson or Spearman correlation coefficients, but due to the low number of samples here, we do not provide those measures. For both the accuracy and F1 score, it is true that high values are not necessarily associated with high ranking (e.g., LadderNet and IterNet in Figure 7a and RVSGAN and SA-UNet in Figure 7b) or that low values are associated with low rankings (e.g., IterNet in Figure 7a and UNet in Figure 7b). This supports the claim that high values for objective metrics do not always correlate with the clinical viability of method results, as was demonstrated in other medical diagnostic fields [15]. In other words: perpixel classifier metrics do not, in this instance, measure network quality as seen from an application standpoint.

The most important results of the second stage of this research are to establish a partial ranking of methods based on the opinion of domain expert observers and to demonstrate that there is no correlation between observer opinion on clinical viability and commonly used objective metrics of network quality. The latter result demonstrates that the objective metrics do not necessarily measure how useful the network output will be in clinical practice. A possible reason for the lack of correlation between objective metrics and diagnostic importance can be that objective metrics treat all image pixels as equally important, while in practice, pixel groups depicting disease indicators are the most important.

## 4. Conclusions

This paper addressed two hypotheses: The first was that it was possible to, using modern CNN models, extract sufficient fundus information into a segmentation mask for a human ophthalmologist to use as the sole material for a diagnosis. This, if demonstrated, would present a path for clinical adoption, and if the clinical quality of various CNNs could be measured, the best candidate or candidates for adoption could be determined.

The experiment to test this hypothesis was conducted by having a team of experienced ophthalmologists attempts to create diagnoses based on various segmentation masks. The results of the test showed low reliability of results and low accuracy of diagnosis. This led us to suspect that the segmentation results were clinically insufficient in the general case and that the test was effectively impossible, demanding from its participants something beyond the limits of clinical possibility.

Therefore, we formulated a secondary hypothesis: namely that, even if the current state of the art is insufficient for immediate deployment, its performance trends upward over time, and steady technological progress will eventually deliver sufficient accuracy. There is, simply put, a natural ranking to the clinical applicability of networks. We expected, furthermore, that this ranking should increase over time and match the values objective metrics provide. To test this hypothesis we measured the rating the team of ophthalmologists gave to various segmentation masks created by various networks operating on various datasets. The expected outcome is that there will be differences in how images are perceived, that these differences in perceived clinical quality will be stable, intra-observer and over time, and that they will track the objective measures of quality.

Tests have shown that differences do, indeed, exist (though with no absolute winner) and that they are quite stable over time. However, they have not been observed to track with objective measures in quality, showing a pattern of anti-correlation.

Therefore, we were forced to conclude that per-pixel objective measures of quality in present use are not fit for purpose when it comes to measuring the effectiveness of this type of CNN and that it is possible that what is being skillfully optimized for in numerous papers does not provide any tangible clinical benefit and may be damaging the effectiveness of the result. Future avenues of work should perhaps focus on multi-network models that allow for more flexible applicability to clinical use and the development of quality metrics more closely aligned to clinical needs over possibly spurious per-pixel accuracy.

## Figures and Tables

**Table 1:** CNN training hyperparameters, as reported in the papers. A value of Unknown is where we were unable to find the information.

Network	Epochs	Batch Size	Learning Rate	Loss	Optimizer
UNet	150	4	1 × 10^−4^	Dice	Adam
DBUNet	250	64	3 × 10^−4^	Weighted mean	Adam
ESWANet	20	4 (mini-batch)	5 × 10^−2^ (with complex decay 1 × 10^−6^)	Cross-entropy	SGD with Nesterov momentum
SA-UNet	100	2	1 × 10^−3^	Binary cross-entropy	Adam
LadderNet	150	1024	1 × 10^−2^ (with decay $10^{-1}$)	Cross-entropy	Adam
IterNet	Unknown	32	1 × 10^−3^	Binary cross-entropy	Adam
RVSGAN	10	Unknown	2 × 10^−4^	Binary cross-entropy	Adam

**Table 2:** A list of fundus images whose corresponding segmentation masks are included in the survey. The table includes the image name that matches what it was named in the publicly available dataset (the first column). The dataset name (the second column), the diagnosis associated with the image according to ground truth values provided by the authors of the corresponding dataset (the third column), and the diagnostic category are listed, as in the survey (the last column).

Image	Dataset	Diagnosis According to the Authors of the Dataset	Diagnosis in This Paper
26_training	DRIVE	Background diabetic retinopathy	Diabetic retinopathy
25_training	DRIVE	Background diabetic retinopathy	Diabetic retinopathy
32_training	DRIVE	Background diabetic retinopathy	Diabetic retinopathy
14_test	DRIVE	Background diabetic retinopathy	Diabetic retinopathy
08_test	DRIVE	Background diabetic retinopathy	Diabetic retinopathy
03_test	DRIVE	Background diabetic retinopathy	Diabetic retinopathy
17_test	DRIVE	Background diabetic retinopathy	Diabetic retinopathy
im0001	STARE	Background diabetic retinopathy	Diabetic retinopathy
im0002	STARE	Arteriosclerotic retinopathy and choroidal neovascularization	Arteriosclerotic retinopathy
im0004	STARE	Cilio-retinal artery occlusion or arteriosclerotic retinopathy	Cilio-retinal artery occlusion or arterosclerotic retinopathy
im0005	STARE	Central retinal artery occlusion and central retinal vein occlusion	Central artery/vein occlusion
im0139	STARE	Background diabetic retinopathy	Diabetic retinopathy

**Table 3:** Per class classification accuracy expressed as a percent of the correct diagnoses per diagnostic class. In parentheses, next to each accuracy, there is an image count per diagnosis in the survey dataset (expressed in percentages of overall images).

Diabetic Retinopathy	Central Retinal Artery/Vein Occlusion	Cilio-Retinal Artery Occlusion	Arteriosclerotic Retinopathy
43.49% (75.0%)	44.29% (16.67%)	2.86% (8.33%)	8.57% (8.33%)

**Table 4:** Reliability analysis measurements. The table displays values of Cronbach’s α with confidence intervals (CI) and Guttman’s $\lambda_{6}$ for grading score and answer correctness measurements aggregated for all observers in the experiment and the most experienced observer separately.

Measure	All Observers			Observer 1		
	α	$\lambda_{6}$	α 95% CI	α	$\lambda_{6}$	α 95% CI
Grading score	0.5	0.3	0.23–0.61	0.6	0.4	0.18–0.83
Correctness	0.4	0.3	0.17–0.58	0.6	0.4	0.17–0.83

**Table 5:** CNN model ranking based on the Copeland score for all pairwise comparisons. The larger the value is, the more significant the model is clinically. Absolute values of the Copeland score are in the [−8,8 ] range.

Dataset/CNN	ESWANet	IterNet	IterNet_UNI	LadderNet	SA-UNet	DBUNet	RVSGAN	UNet
All	1	−3	−5	4	1	−7	5	4

**Table 6:** CNN model ranking based on the Copeland score for each of the datasets. The larger the value is, the more significant the model is clinically. Absolute values of the Copeland score are in the [−8, 8] range.

Dataset/CNN	ESWANet	IterNet	IterNet_UNI	LadderNet	SA-UNet	DBUNet	RVSGAN	UNet
STARE	3	−4	0	4	−6	4	0	−1
CHASE	0	−5	−7	−2	3	3	7	1
DRIVE	2	2	0	1	3	−7	−1	0

**Table 7:** CNN model ranking based on the Copeland score for each of the observers. The larger the value is, the more significant the model is clinically. Absolute values of the Copeland score are in the [−8, 8] range.

Observer/CNN	ESWANet	IterNet	IterNet_UNI	LadderNet	SA-UNet	DBUNet	RVSGAN	UNet
Observer 1	5	−3	1	−3	−5	−5	5	5
Observer 2	−1	−5	−5	3	7	−5	3	3
Observer 3	3	7	−3	−3	−1	−7	3	1
Observer 5	5	−5	−5	7	−1	−1	1	−1

**Figure A1:**
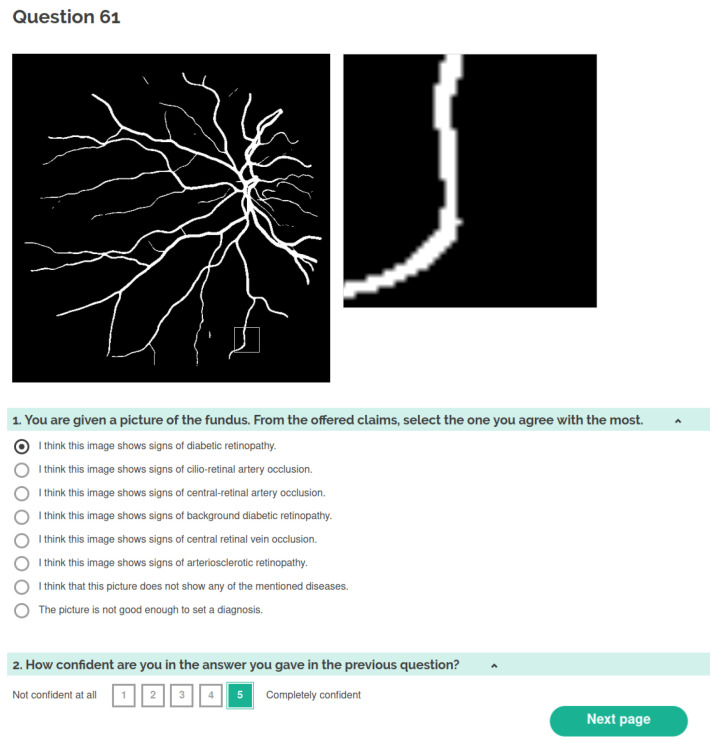
A representative page of the survey used in the first experiment with examples of selected answers.

**Figure A2:**
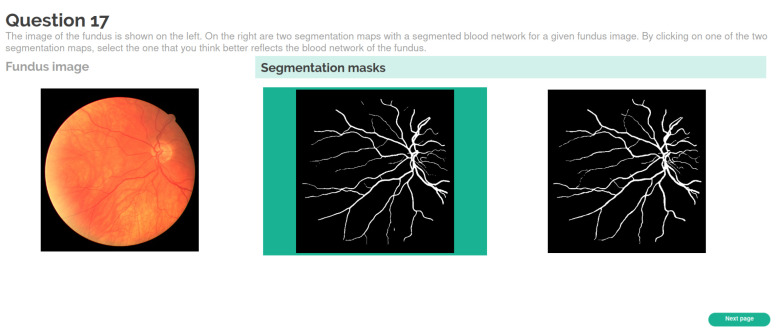
A representative page of the survey used in the second experiment with examples of the selected answer.

## Data Availability

The source code of the survey generator and generated surveys can be found at https://github.com/goranagojic/survey-generator (accessed on 16 November 2022). The source code of data curation and analysis software, as well as survey results, can be found at https://github.com/goranagojic/retina-subjective-assessment (accessed on 16 November 2022). A lightweight, interactive notebook to reproduce paper results is available at https://bit.ly/3VOyBSC (accessed on 16 November 2022).

## Abbreviations

    The following abbreviations are used in this manuscript:

AI	Artificial Intelligence
CHASE	Child Hearth and Health Study in England
CNN	Convolutional Neural Network
CT	Computed Tomography
DRIVE	Digital Retinal Images for Vessel Extraction
GAN	Generative Adversarial Networks
FoV	Field of View
MRI	Magnetic Resonance Imaging
MCC	Matthews Correlation Coefficient
STARE	Structured Analysis of Retina

## Appendix A. Survey Design Choices

This appendix serves to discuss the survey design details in more depth rather than burden the main body of text. In Figure A1, we show a page from a survey presented to the observers in the first experiment. The largest image on the left shows one segmentation mask whose effectiveness for clinical purposes is being assessed. To assure that the observer can see the details in the segmentation mask regardless of the screen size they use during evaluation, the survey implements a zoom-on-hover feature, enabling the observer to enlarge a part of the segmentation mask by placing the cursor above it. The zoomed-in part of the image is shown in the smaller of the two images in Figure A1. The two questions related to the image are next. In the first one, the observer chooses a diagnosis they would associate with the shown segmentation mask or marks the image as insufficient quality to set a diagnosis. Alternatively, the observer might assess that the segmentation mask corresponds to a healthy eye or shows signs of disease not listed in the choices. In that case, they can opt for a ’none of the above’ choice. The first question is implemented in the form of a radio button group selection, so the observer must opt for just one of the choices they are presented with. Here the user is presented with original diagnoses from the dataset and not the aggregated diagnoses used in the analysis. By offering higher levels of granularity, it is possible to perform data analysis on a finer level while still preserving the possibility of analyzing the aggregated data. If the user selects ’background diabetic retinopathy’ (finer clinical nomenclature) or ’diabetic retinopathy’, the answer is interpreted as belonging to the aggregated ’diabetic retinopathy’ class all the same. Similarly, the user is presented with different choices for central retinal artery and vein occlusion to test if the observer can differentiate between the two just based on blood vessel position in the image without explicit artery/vein labels.

In the second question, the observer is asked to express their confidence in response to the first question by selecting a value on a 5-level Likert scale, where value 1 stands for ’not confident at all’ while value 5 stands for ’completely confident’. The values of the Likert scale are arranged from left to light, most to least confidence, as is typical. To proceed to the next question, the observer must answer both questions.

Figure A2 shows one page from the survey used in the second experiment. Here, the observer is presented with three images—the leftmost is an unmodified color fundus image, while the other two are two segmentation masks produced by different methods for the displayed fundus image. The observer is asked to select one of the two segmentation masks they find better from a clinical viability point of view. The observer is required to select one of the two segmentation masks before proceeding to the next question.

## Appendix B. Pseudo-Code to Calculate a Copeland Score

In this section, we show the pseudo-code that describes an algorithm to compute the Copeland score used for CNN ranking in the result analysis of the second survey results. The algorithm is intended to help readers understand the details of survey data manipulation required to produce a Copeland score for a set of eight segmentation masks for a single fundus image. The paper software repository contains an implementation of Algorithm A1 in real code form.

**Algorithm A1** Copeland score calculation.
Require: Survey data where each entry is a tuple $(candidate_{1}$, $candidate_{2}$, winning_candidate). Each candidate is one of the segmentation masks involved in the survey. Iwinning_candidate is one of the $candidate_{1}$ or $candidate_{2}$ images. *% An algorithm to calculate the Copeland score based on candidate pairwise comparisons %* 1: **function** calculateCopelandScore(survey_data) 2: candidates← eight segmentation masks for the same fundus image 3: candidate_pairs←getCombinations(candidates) 5: ranking_table←empty_table 6: **for** candidate in candidates **do** 7: copeland_score←0 8: ranking_table.insert(candidate, copeland_score) 9: **end for** 11: **for** $\left(c_{1},\;c_{2}\right)$ in candidate_pairs **do** 12: $c_{1}\_wins\leftarrow countWins\left(candidate=c_{1}\right)$ 13: $c_{2}\_wins\leftarrow countWins\left(candidate=c_{2}\right)$ 14: **if** $c_{1}\_wins>c_{2}\_wins$ **then** 15: $ranking\_table.where(candidate=c_{1}).copeland\_score\;+=\;1$ 16: **else if** $c_{1}\_wins<c_{2}\_wins$ **then** 17: $ranking\_table.where(candidate=c_{1}).copeland\_score\;-=\;1$ 18: **end if** 19: **end for** 20: **end function** *% Counts how many times has candidate won in pairwise comparisons according to survey data stored in survey_entries. %* 21: **function**countWins(survey_entries, candidate) 22: counter←0 23: **for** entry **in** survey_entries **do** 24: $c_{1}\leftarrow entry.candidate_{1}$ 25: $c_{2}\leftarrow entry.candidate_{2}$ 26: **if** candidate **in** ($c_{1},\;c_{2}$) **then** 27: **if** candidate **is** entry.winning_candidate **then** 28: counter += 1 29: **end if** 30: **end if** 31: **end for** 32: **return** counter 33: **end function** *% Get all combinations of unique candidate pairs. That is 24 for 8 segmentation images. %* 34: **function**getCombinations(candidates) 35: n←8 36: combinations←empty_list 37: **for** *i* **in** (0 **to** n) **do** 38: $c_{1}\leftarrow candidates.at\_position\left(i\right)$ 39: **for** *j* **in**(0 **to** n) **do** 40: $c_{2}\leftarrow candidates.at\_position\left(j\right)$ 41: $combinations.insert(\left(c_{1},\;c_{2}\right))$ 42: **end for** 43: **end for** 44: **end function**
